# Supplementary material for: Genome-wide identification of Aedes albopictus long noncoding RNAs and their association with dengue and Zika virus infection
Source: PLoS Negl Trop Dis. 2021 Jan 22;15(1):e0008351. doi: 10.1371/journal.pntd.0008351 (PMC7872224; doi:10.1371/journal.pntd.0008351)
Supplement: S3 Table — (DOCX) [file pntd.0008351.s003.docx]

| Sample | Accession number | Total reads | Mapped reads | Mapping rate (%) |
| --- | --- | --- | --- | --- |
| Mock replicate 1 | SRR10123563 | 53887179 | 38410781 | 71.28 |
| Mock replicate 2 | SRR10123558 | 56106951 | 40441890 | 72.08 |
| Mock replicate 3 | SRR10123557 | 48009966 | 34821628 | 72.53 |
| ZIKV replicate 1 | SRR10123561 | 44665232 | 32105369 | 71.88 |
| ZIKV replicate 2 | SRR10123560 | 42353023 | 30286647 | 71.51 |
| ZIKV replicate 3 | SRR10123559 | 42059785 | 30943384 | 73.57 |
| DENV-1 replicate 1 | SRR10123556 | 52414565 | 41978825 | 80.09 |
| DENV-1 replicate 2 | SRR10123555 | 43731890 | 33345566 | 76.25 |
| DENV-1 replicate1 3 | SRR10123562 | 43227204 | 37331013 | 86.36 |
