# Supplementary material for: Genome-wide identification of Aedes albopictus long noncoding RNAs and their association with dengue and Zika virus infection
Source: PLoS Negl Trop Dis. 2021 Jan 22;15(1):e0008351. doi: 10.1371/journal.pntd.0008351 (PMC7872224; doi:10.1371/journal.pntd.0008351)
Supplement: S8 Table — (DOCX) [file pntd.0008351.s008.docx]

**qPCR primers**

| **Primers** | **Sequence** | **Size of PCR product (bp)** |
| --- | --- | --- |
| lncRNA_1008.1 forward | CGCAACGAATTTATAATCAGTGAGA | 242 |
| lncRNA_1008.1 reverse | GCGAATGATGATGATGCTCGATG |  |
| XR_003895025.1 forward | AGTTTCGAATAAAACCATCATGGAG | 249 |
| XR_003895025.1 reverse | CGAAGTGGGTACGCTGGTAA |  |
| lncRNA_13375.1 forward | TCAACGTTTGGATGGATGGAGA | 224 |
| lncRNA_13375.1 reverse | AAAGCCATCACCCTGCCAAT |  |
| lncRNA_27639.2 forward | TGTGGATCTCCACCCGTACT | 179 |
| lncRNA_27639.2 reverse | GAACCCCATAGTGGGGAAGC |  |
| XR_003899061.1 forward | ACTCGTGTTGATTAGTCGGACC | 219 |
| XR_003899061.1 reverse | TACTCAATGACGCCCCACG |  |
| lncRNA_46959.1 forward | TCGCTCTGATTTGAACGGCT | 163 |
| lncRNA_46959.1 reverse | TGGAGGGTTCAAAAAGTCGGT |  |
| lncRNA_50256.1 forward | TGCTTCGTCCCGGAATCATAA | 173 |
| lncRNA_50256.1 reverse | ACCGAAACGGAACGGGAAC |  |
| XR_003892459.1 forward | TGTGCGTTTTCTCACCAGGA | 214 |
| XR_003892459.1 reverse | TGTTGAGCCTGAAGCGATGT |  |
| lncRNA_56589.2 forward | ATTCGCAACTCCCCAACCAT | 169 |
| lncRNA_56589.2 reverse | GCAATTTCCCGCACGTAGTC |  |
| lncRNA_59941.1 forward | GGCGGTGTAGTGTGAGGTTA | 179 |
| lncRNA_59941.1 reverse | TCCATGGCTAGTGTGACTGT |  |
| lncRNA_6455.1 forward | GTGCCACGGCGTAGAAGATA | 157 |
| lncRNA_6455.1 reverse | CGTCCTGCTGTCTGTAGCAA |  |
| lncRNA_27080.54 forward | CCACCGTCGCGTCTGACTA | 221 |
| lncRNA_27080.54 reverse | CGCGTCAGAATGTGAACTGC |  |
| XR_003896399.1 forward | TCAGCAAAGTGCGCTGGATA | 127 |
| XR_003896399.1 reverse | AGTTCCTCACACGCGACAAT |  |
| lncRNA_3250.2 forward | GGACTGGTCACCTAACGACG | 174 |
| lncRNA_3250.2 reverse | GAAGGAGATCAGCCCTCGTG |  |
| XR_002132174.2 forward | CTTGTTTGGATCGGAACGGC | 101 |
| XR_002132174.2 reverse | TGGATGTGACGAACGGGAAG |  |
| lncRNA_22669.1 forward | TTTTGCGCTAGCCAAGCAAG | 250 |
| lncRNA_22669.1 reverse | GCCGTATGGCATTCATGGTG |  |
| LOC109431891 forward | TTGCAAGCCCCATACTAGCC | 208 |
| LOC109431891 reverse | TTCCCGACTGGACTCCTTCT |  |
| LOC115269840 forward | ATGTCGATGCTGTGGAAACG | 141 |
| LOC115269840 reverse | ATCGTAAATGTGTTCCCGCG |  |

**gRNA sequences**

| **Primer name** | **Sequence (5’-3’)** |
| --- | --- |
| lncRNA_27639.2 gRNA_F | ttcGGATGGCTCGACCTTCAGTA |
| lncRNA_27639.2 gRNA_R | aacTACTGAAGGTCGAGCCATCC |
| XR_003899061.1_gRNA_F | ttcgTCTGATTCCAAGCGCTGGTA |
| XR_003899061.1_gRNA_R | aacTACCAGCGCTTGGAATCAGAc |

**PCR primers flanking the cleavage site**

| **Primers** | **Sequence** | **Size of PCR product (bp)** |
| --- | --- | --- |
| lncRNA_27639.2 left | ACCCCAATGACAATTTCTTTCA | 225 |
| lncRNA_27639.2 right | TTGAACAATCCTGAGTGTCCTGA |  |
| XR_003899061.1 left | TTGATTAGTCGGACCGAGATTT | 204 |
| XR_003899061.1 right | AACTTACCACGCGGATCAATAA |  |
