# Supplementary figures and images for: Genome-wide identification of Aedes albopictus long noncoding RNAs and their association with dengue and Zika virus infection
Source: PLoS Negl Trop Dis. 2021 Jan 22;15(1):e0008351. doi: 10.1371/journal.pntd.0008351 (PMC7872224; doi:10.1371/journal.pntd.0008351)

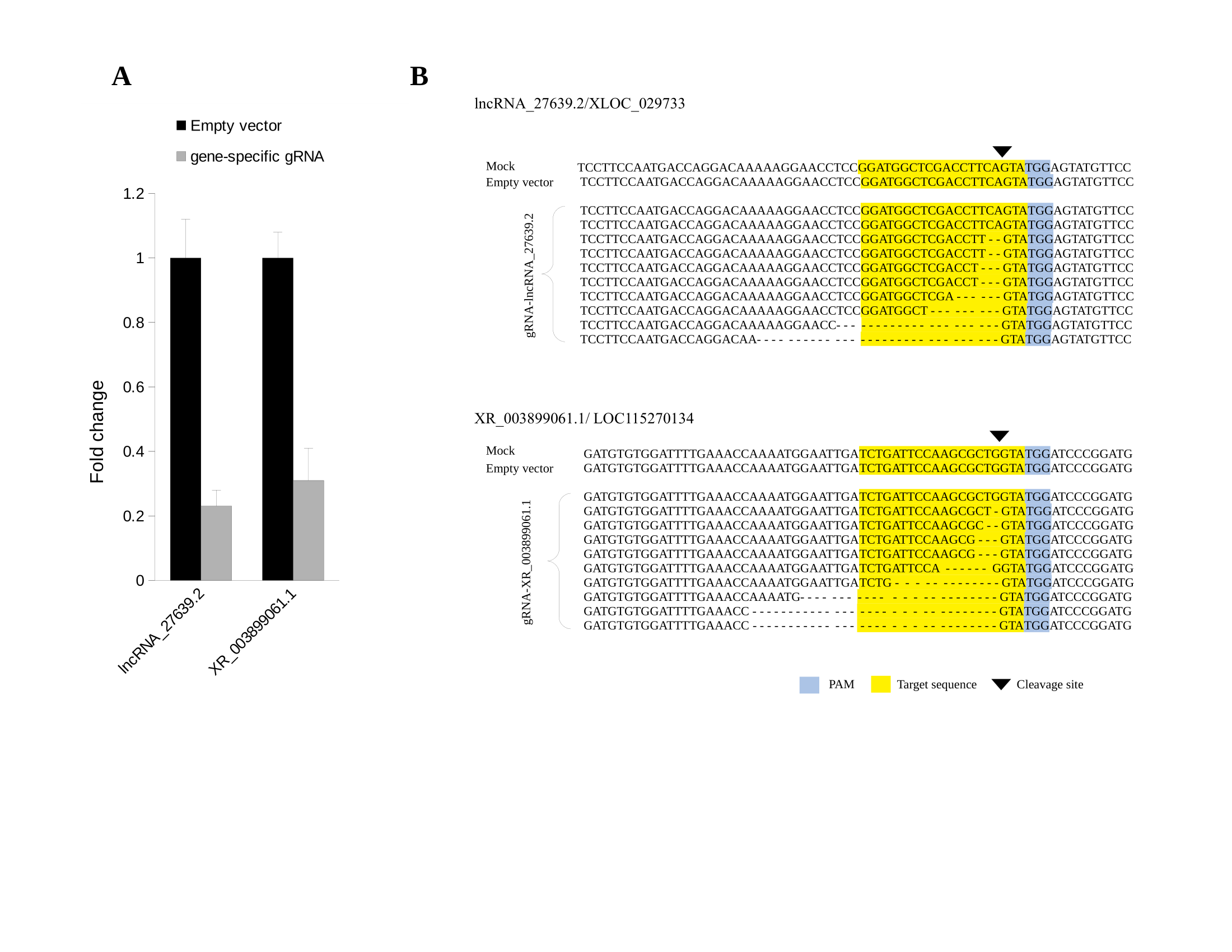

Supplement: S3 Fig — A) As determined by RT-qPCR, CRISPR-Cas9 induced mutation resulted in lower expression level of lncRNA_27639.2 and XR_003899061.1 in C6/36 cells. Expression levels were normalized to empty vector sample. All changes in expression level shown in the figure are statistically significant with P-value < 0.05. Bars indicate mean +/- SEM of three independent experiments. Student’s t-test was used for all comparisons. B) Sequencing of indel mutations within lncRNA loci after 5 days of puromycin selection. PCR products flanking the cleavage site (black triangle) were cloned and sequenced from C6/36 cells transfected with vector containing lncRNA-specific sgRNAs. Majority of the clones showed deletions at the expected cleavage sites. Empty vector refers to the Drosophila CRISPR-Cas9 vector (pAc-dU6-sgRNA-Cas9) without gRNA targeting any gene. In mock sample, the C6/36 cells were transfected with transfection reagent only (Lipofectamine 2000). In both mock and empty vector samples, the clones showed wild type sequence. PAM is highlighted in blue, while target sequence in yellow. (TIFF) [file pntd.0008351.s011.tiff]
